# Complete mitochondrial genome of the Volk’s sculpin *Cottus volki* (Cottoidei: Cottidae)

**DOI:** 10.1080/23802359.2017.1307705

**Published:** 2017-03-29

**Authors:** Evgeniy S. Balakirev, Pavel A. Saveliev, Francisco J. Ayala

**Affiliations:** a Department of Ecology and Evolutionary Biology, University of California, Irvine, CA, USA;; b A.V. Zhirmunsky Institute of Marine Biology, National Scientific Center of Marine Biology, Far Eastern Branch, Russian Academy of Sciences, Vladivostok, Russia;; c School of Natural Sciences, Far Eastern Federal University, Vladivostok, Russia

**Keywords:** Volk’s sculpin *Cottus volki*, intraspecific divergence, sedentary lifestyle, Cottidae

## Abstract

The complete mitochondrial genome was sequenced in two individuals of the Volk’s sculpin *Cottus volki*. The genome sequences are 16,519 and 16,536 bp in size, and the gene arrangement, composition, and size are very similar to the other sculpin mitochondrial genomes published previously. The difference between the two genomes studied is relatively high, 3.42%, which is 30% of the average interspecific divergence (8.76%) detected between seven *Cottus* species from the GenBank database. The data are consistent with the sedentary lifestyle in *C. volki*, limiting gene flow even between neighbouring rivers.

The Volk’s sculpin *Cottus volki* Taranetz 1933 is a freshwater fish inhabiting the Sea of Japan’s inland coastal rivers (Taranets 1933; Shedko 2001). Berg (1949) as well as Sideleva (2002) has considered *C. volki* to be a subspecies of the spotted sculpin *C. poecilopus* Haeckel. Later, based on the short mitochondrial (mt) sequences (control region) and morphology, the validity of *C. volki* was established (Shedko & Miroshnichenko 2007; Yokoyama et al. 2008; Sideleva & Goto 2009). However, the complete mt genome of *C. volki* had not yet been sequenced to confirm the species identity and to clarify its relationships with the other species belonging to the genus *Cottus*.

We have sequenced two complete mt genomes of *C. volki* (GenBank accession numbers KY563343 and KY563344) from the Alekseevka river (43° 65′ 08 N; 133°51′ 44 E) and Zerkal'naya river (44° 26′ 77 N; 135° 27′ 60 E), Primorsky krai, Russia, using primers designed with the program mitoPrimer_V1 (Yang et al. 2011). The fish specimens are stored at the museum of the A. V. Zhirmunsky Institute of Marine Biology, National Scientific Center of Marine Biology, Vladivostok, Russia (www.museumimb.ru) under accession numbers MIMB 33260 and MIMB 33261. The mt genome sequences are 16,519 and 16,536 bp in size; the gene arrangement, composition, and size are very similar to the sculpin fish genomes published previously. There are 564 single nucleotide and 14 length differences (some involving more than one bp) between the two haplotypes of CVO45 and CVO61; total sequence divergence (*D*_xy_) is 0.0342 ± 0.0014.

Comparison of the two mt genomes now obtained with other complete mt genomes available in GenBank for the genera *Cottus* and *Mesocottus* reveals a close affinity of *C. volki* to the cluster of *C. czerskii* +* C. szanaga* within the genus *Cottus* (Figure 1). The difference (*D*_xy_) between *C. volki* and the cluster *C. czerskii* +* C. szanaga* is 0.0707 ± 0.0012, which is in close agreement with the average divergence between the *Cottus* species from the GenBank database (0.0846 ± 0.0014).

**Figure 1.**
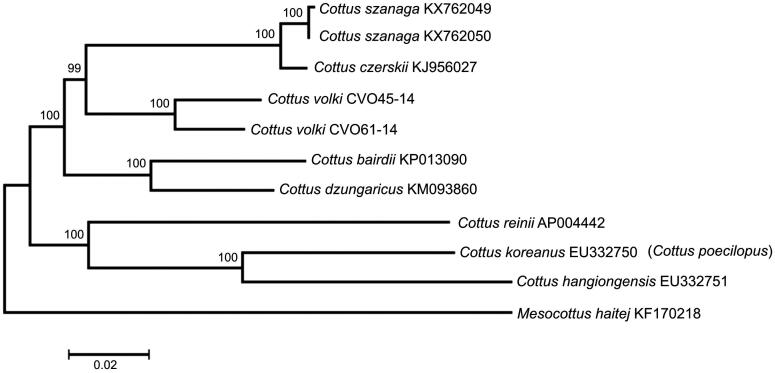
Maximum-likelihood tree for the Volk’s sculpin *C. volki* specimens CV045-14 and CVO61-14, and GenBank representatives of the family Cottidae. The tree is constructed using whole mitogenome sequences. The tree is based on the General Time Reversible + gamma + invariant sites (GTR + G + I) model of nucleotide substitution. The numbers at the nodes are bootstrap per cent probability values based on 1000 replications. The GenBank species name for the accession number EU332750 is in parentheses.

The difference between the two *C. volki* mt genomes now studied is relatively high, 3.42%, which is only 2.56 times less (30% of it) than the average interspecific divergence, 8.46%, detected between all seven *Cottus* mt genomes available in GenBank, including *C. bairdii*, *C. czerskii*, *C. dzungaricus*, *C. hangiongensis*, *C. koreanus*, *C. reinii*, and *C. szanaga*. The data are consistent with the sedentary lifestyle of *C. volki*, limiting gene flow even between neighbouring rivers (Kolpakov 2011).
